# Dietary and *Plasmodium* challenge effects on the cuticular hydrocarbon profile of *Anopheles albimanus*

**DOI:** 10.1038/s41598-021-90673-x

**Published:** 2021-05-27

**Authors:** Fabiola Claudio-Piedras, Benito Recio-Tótoro, Jorge Cime-Castillo, Renaud Condé, Massimo Maffei, Humberto Lanz-Mendoza

**Affiliations:** 1 Centro de Investigaciones Sobre Enfermedades Infecciosas, Instituto Nacional de Salud Pública, 62300 Cuernavaca, Morelos Mexico; 2 Instituto de Biotecnología, Universidad Nacional Autónoma de México, 62300 Cuernavaca, Morelos Mexico; 3 Dipartimento di Scienze della Vita e Biologia dei Sistemi, Università degli Studi di Torino, 10135 Torino, Italy

**Keywords:** Malaria, Lipidomics

## Abstract

The cuticular hydrocarbon (CHC) profile reflects the insects’ physiological states. These include age, sex, reproductive stage, and gravidity. Environmental factors such as diet, relative humidity or exposure to insecticides also affect the CHC composition in mosquitoes. In this work, the CHC profile was analyzed in two *Anopheles albimanus* phenotypes with different degrees of susceptibility to *Plasmodium*, the susceptible-White and resistant-Brown phenotypes, in response to the two dietary regimes of mosquitoes: a carbon-rich diet (sugar) and a protein-rich diet (blood) alone or containing *Plasmodium* ookinetes. The CHCs were analyzed by gas chromatography coupled to mass spectrometry or flame ionization detection, identifying 19 CHCs with chain lengths ranging from 20 to 37 carbons. Qualitative and quantitative changes in CHCs composition were dependent on diet, a parasite challenge, and, to a lesser extent, the phenotype. Blood-feeding caused up to a 40% reduction in the total CHC content compared to sugar-feeding. If blood contained ookinetes, further changes in the CHC profile were observed depending on the *Plasmodium* susceptibility of the phenotypes. Higher infection prevalence caused greater changes in the CHC profile. These dietary and infection-associated modifications in the CHCs could have multiple effects on mosquito fitness, impacts on disease transmission, and tolerance to insecticides.

## Introduction

The cuticle is the most extensive extracellular structure covering the insect’s external surfaces, protecting them against the environment. It is mainly composed of chitin, proteins, lipids, waxes, and cement. The cuticle covers all the body parts exposed to the external environment, including cavities, the foregut and hindgut coatings, the luminal side of the trachea and tracheoles, as well as the wings. Therefore, the cuticle is the interface between the external and the internal milieu of the insect. On the one hand, it is the point of direct contact with the potentially hostile or challenging exterior, and on the other hand, the cuticle rests on a monolayer of epidermal cells that extends throughout the body of the mosquito, which in turn, is in close contact with the hemolymph through a thin basal lamina. The cuticle is formed as a product of: (i) the active secretion of the underlying epidermal cells^1^, (ii) the prominent secretion of oenocytes located within or under the integument^2^, and (iii) the arrival of nutrients, principally derived from the fat body, through the hemolymph^3,4^.

The cuticle composition is dynamic and changes depending on the age, developmental stage, and metabolic status of the insect^1,2^, exhibiting its essential function in the adaptation capacity and survival of the insect to changing environmental factors^4–6^. Thus, the cuticle’s composition is, in a way, the reflex of the insect’s physiological state. As a multifunctional structure, the cuticle participates in locomotion, desiccation resistance^3^, defence against pathogens and toxins^7,8^, insecticide resistance^4^, and other mechanical and chemical insults.

The cuticle is comprised of multiple layers with different compositions and properties: (1) endocuticle, (2) exocuticle, (3) cuticuline, (4) cerosa, and (5) cement^9^. The endocuticle and exocuticle form in conjunction a region commonly named procuticle, which represents the major portion of the total cuticle and is composed mainly of chitin, proteins, lipids, salts, and pigments. The cuticuline, cerosa, and cement comprise the most external layer known as epicuticle, rich in extractable lipids and a complex mixture of hydrocarbons, fatty acids, and waxes^7,10^. In most insects, the cuticular hydrocarbons (CHCs) include n-alkanes, methy-branched alkanes, and alkenes. The CHCs studied until recently have shown functions related to desiccation resistance, as mating signals, species and gender recognition cues, nestmate recognition, dominance and fertility cues, chemical mimicry, and for preventing infections^3–5,7,10,11^.

In *Anopheles* mosquitoes, the CHCs have been widely used for identifying species such as *An. gambiae*^2,12–14^, *An. culicifacies*^15^, *An. maculipennis*^16^, *An. maculatus*^17^, *An. darlingi*^18^, *An. stephensi*^19^, *An. quadrimaculatus*^20^, *An. claviger*, among others^21^. The analysis of the CHCs composition in anopheline mosquitoes has been made mainly from cuticular extracts with n-hexane and analyzed by gas chromatography-mass spectrometry coupled with multivariate analysis^10^, confirming that the principal CHCs in mosquitoes are n-alkanes with chain lengths ranging from 15 to 47 carbons (C15–C47), followed to a lesser extent by alkenes^14,15,17,20,22–24^. The primary reported function for the n-alkanes is control of transcuticular water movement. At the same time, the unsaturated compounds and the methyl-branched CHCs, are more likely to be involved in communication^3^.

A notorious aspect of the characterization of CHCs in anopheline mosquitoes is their utility in distinguishing between sibling species from different species complexes and karyotypes^12,13,15,18,19,21,25,26^, the determination of age, sex, and reproductive status^1,5,14,22,23,27^, of vector or non-vector species^17,21^, and insecticide-resistant phenotypes^4,24,28,29^. However, despite being vectors of the parasite that causes malaria, little is known about the CHCs content or the cuticle's modifications after blood feeding and during a *Plasmodium* infection.

Female mosquitoes must feed on blood to initiate ovary development and lay eggs. This is also when the malaria parasites are transmitted from host to vector and vice versa. After a mosquito feeds on blood-containing gametocytes, the latter mature into gametes and fertilization occurs. The resulting zygotes differentiate into ookinetes that invade the mosquito’s midgut epithelium to transform into oocysts. With the support of nutrients from the mosquito, the oocysts grow in size from 5 to about 60 µm in diameter, forming thousands of sporozoites that are freed to the hemolymph. The sporozoites then reach the salivary gland and invade it to be transmitted to new hosts upon subsequent feedings of the mosquito^30^.

*An. albimanus* is one of the principal human malaria vectors in Central America, the northern part of South America, and the Caribbean^31^. The Tapachula strain of *An. albimanus* was initially collected on the Coastal Plain of southern Chiapas-Mexico, and presents two predominant phenotypic variants^32^. These variants are distinguishable by the presence or absence of a morphological marker denominated *stripe*^33^, a white stripe visible in the dorsal side of the larvae and pupae. Mosquitoes lacking this marker present a homogenous brown coloration. The first observations related to these phenotypes showed that the *stripe* marker is dominantly inherited over the phenotype without the marker^34^, where the genetic determinant is thought to reside in chromosome 3^35^. The first works regarding the association between the phenotypic variants with physiological characteristics in *An. albimanus* were developed around the phenomenon of insecticide and infection resistance^33,36^. The White (*stripe*^+^) phenotype mosquitoes are resistant to dieldrin while more susceptible to infections with *Plasmodium vivax* and *P. berghei* compared to the Brown (*stripe*^*−*^) phenotype^37,38^.

Furthermore, the stripe’s presence has been associated with higher uric acid content in the larvae^39^. We have recently reported additional differences between the phenotypes at a transcriptional level and in the content of genomic 5-methylcytosine^38^. These differences between the White phenotype in comparison to the Brown phenotype consist in: (i) a lower genomic 5-methylcytosine content, (ii) lower basal transcriptional activity, and (iii) lower transcriptional response to a *P. berghei* challenge. In this study, we analyzed the cuticular CHCs in two phenotypes of *An. albimanus* as an effort to further characterize the physiological differences associated with the susceptible/resistance condition. We show that the CHCs composition of *An. albimanus* mosquitoes is diet-dependent and that a *P. berghei* infection alters its composition, especially in the infection-susceptible phenotype, where the CHC composition changes more drastically. This work provides new information to investigate the potential use of CHCs to distinguish *Plasmodium* infected mosquitoes and opens new perspectives for the understanding of the molecular mechanism in the ecological triad between parasites, ambient, and vectors.

## Results

**Figure 1:**
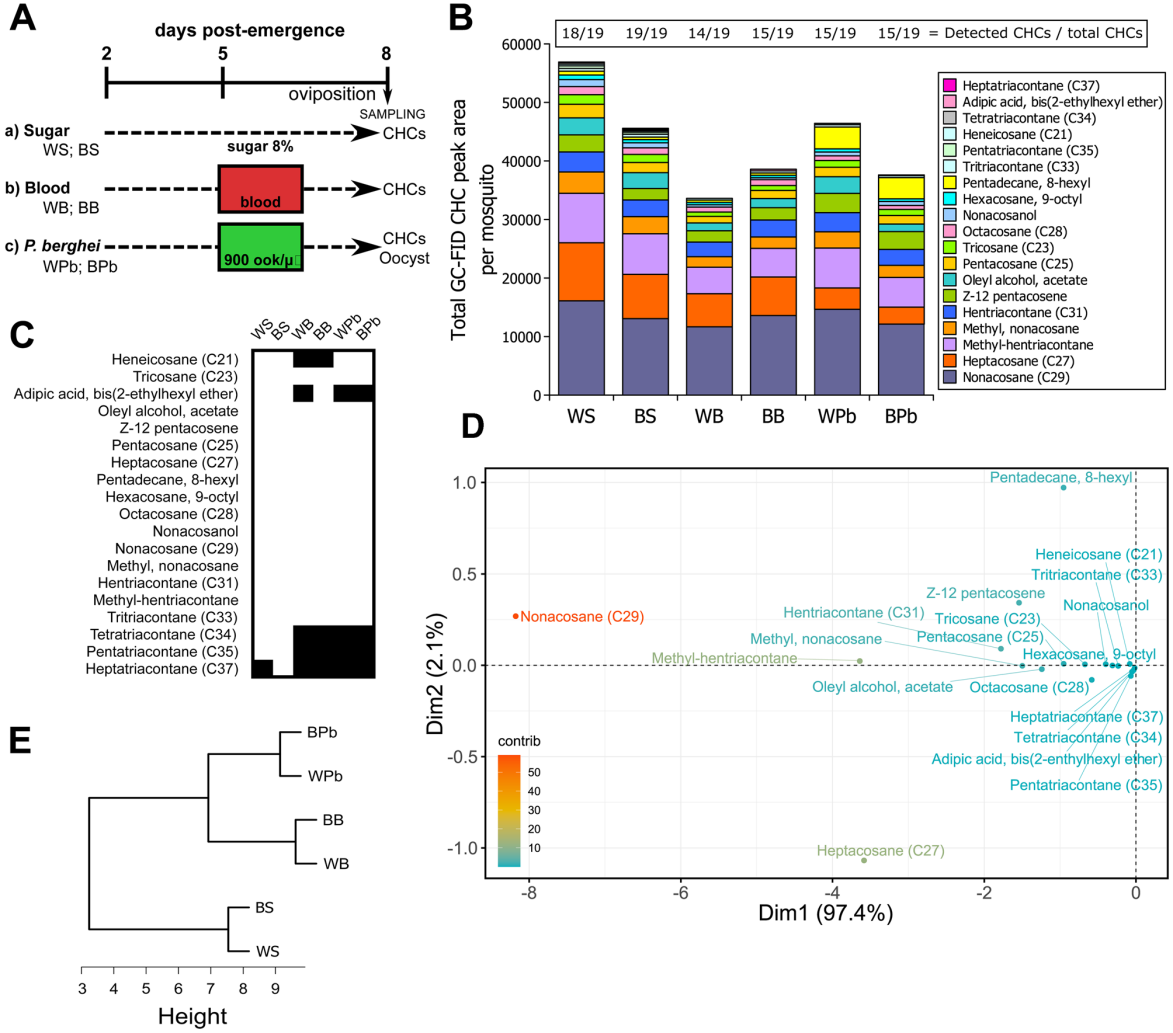
The composition of cuticular hydrocarbons in *An. albimanus* mosquito is diet and infection dependent. (**A**) Experimental setup for the determination of the CHC composition and abundance in the susceptible (White) and resistant (Brown) to infection phenotypes. White (W) and Brown (B) female mosquitoes were fed on 8% sucrose solution (WS; BS) for the entire experiment. Additionally, at 5 days post-emergence (dpe) mosquitoes were, blood-fed (WB; BB), or fed with 900 *P. berghei* ookinetes per microlitre (WPb; BPb). CHC extraction was carried out three days post feeding from 50 mosquitoes per group in triplicates for GC identification. Oocyst counts per mosquito were also determined at eight dpe. (**B**) Stacked area of each CHC per mosquito-equivalent (i.e. divided by the number of mosquitoes per sample) normalized by a heptadecane internal standard in the two phenotypes fed with different diets and *Plasmodium*-challenged. Results of two independent experiments performed in triplicates. The number of identified compounds per phenotype and feeding treatment are shown above each column. (**C**) Qualitative changes in the CHC profiles in the two phenotypes fed with the different diets and *Plasmodium*-challenged. The boxes show the presence (white) or absence (black) of the CHC in the mosquito extracts. (**D**) Principal component analysis (PCA) of the mosquitoes’ CHC profiles fed with different diets and *Plasmodium*-challenged. The PCA plot indicates the influence of each CHC in explaining the variation between the phenotypes and feeding groups. The colour gradient (contrib.) indicates the percentage contribution of a compound on the variability of the data. (**E**) Cluster dendrogram of the CHC profiles per phenotype and feeding group. The PCA’s distance matrix comprised of the CHC profiles of the feeding treatments and phenotypes were analyzed to display the hierarchical relationships. The mosquitoes were clustered on basis of their feeding treatments, confirming that the diet induces greater changes in the CHC profiles than the differences the phenotypes display between each other.

To assess whether the CHCs composition in the infection-susceptible White (W) and –resistant Brown (B) phenotypes changed with diet and challenged status, the mosquitoes were separated into three different groups per phenotype: sugar-fed (S), blood-fed (B), and *P. berghei* (Pb)-challenged (Fig. 1A). Mosquitoes were maintained with sugar solution throughout the experiment in addition to the ookinete or mock culture feedings at five days post-emergence (dpe) of the blood-fed and Pb-challenged groups. The mosquitoes were left to lay eggs and at eight dpe, mosquito samples were taken to analyze the CHCs and the infection parameters.

### Changes in the cuticular hydrocarbon profile are diet and infection dependent

The CHC profile of each phenotype submitted to different diets and exposure to the parasite was determined by gas chromatography (GC) coupled to either mass spectrometry (MS) for qualitative analysis, or a flame ionization detector (FID) for quantitative determination. The qualitative analysis identified 19 different CHCs (Table 1). Neither phospholipids nor acylglycerols were detected, indicating that only cuticular and not epidermal compounds were extracted^10^. The CHC composition was different between the phenotypes and feeding treatments. The sugar-fed Brown (BS) phenotype extracts contained all 19 CHCs detected, whereas in the sugar-fed White (WS) phenotype extracts, heptatriacontane (C37) was not detected (Fig. 1B, Table 1). The blood-feeding and parasite challenge caused a decrease in the total number of CHCs. The blood-fed White (WB) mosquitoes presented 14 out of 19 CHCs, and the blood-fed Brown (BB) mosquitoes, Pb-challenged Brown (BPb), and White (WPb) mosquitoes showed 15 out of 19 (Fig. 1B). In the blood-fed White (WB) phenotype extracts, the presence of heneicosane (C21), adipic acid, bis(2-ethylhexyl ether), tetratriacontante (C34), pentatriacontane (C35), and heptatriacontane (C37) was not detected. Almost the same results were obtained from the blood-fed Brown (BB) phenotype extracts, with the sole exception of the presence of adipic acid, bis(2-ethylhexyl ether). Challenging both Brown (BPb) and White (WPb) mosquitoes with *P. berghei* ookinetes caused the drastic reduction to undetectable values of adipic acid, bis(2-ethylhexyl ether), tetratriacontane (C34), pentatriacontane (C35), and heptatriacontane (C37) with respect to the sugar diet (Table 1, Fig. 1B).These qualitative changes are summarized in Fig. 1C, where the absence of a specific CHC from a given feeding treatment is depicted as a black box. Interestingly, blood-feeding and the parasite challenge result in the complete absence of long chain CHCs in both phenotypes (Fig. 1C). These observations indicate a common metabolic response among the phenotypes and feeding/challenged status. The blood and infected blood feedings also caused the absence of short chain CHC’s, specifically heneicosane (C21) in the blood-fed phenotypes, and adipic acid, bis(2-ethylhexyl ether) in the parasite-challenged phenotypes. However, the latter was also absent in the White blood-fed mosquitoes.

**Table 1:** Cuticular hydrocarbons and their abundance found in the White and Brown phenotypes fed with sugar, blood, or ookinete-containing blood (GC-FID peak area per mosquito equivalent).

Cuticular hydrocarbons	White sugar	Brown sugar	White blood	Brown blood	White *P. berghei*	Brown *P. berghei*
	Mean ± SE	Mean ± SE	Mean ± SE	Mean ± SE	Mean ± SE	Mean ± SE
Heneicosane (C21)	304.0 ± 103.8	**238.1 ± 238.1**	ND ± ND	ND ± ND	230.6 ± 18.3	**111.1** ± **111.1**
Tricosane (C23 )	1642.4 ± 473.0	1385.0 ± 1.9	738.2 ± 3.7	845.7 ± 167.3	1156.9 ± 68.4	1038.0 ± 108.5
Adipic acid, bis(2-ethylhexyl ether)	**107.7** ± **107.6**	**141.4** ± **141.4**	ND ± ND	281.8 ± 71.5	ND ± ND	ND ± ND
Oleyl alcohol, acetate	2903.3 ± 389.6	2716.9 ± 186.8	1333.3 ± 169.1	1572.6 ± 1239.4	2844.0 ± 277.9	1280.7 ± 384.6
Z-12 pentacosene	2927.8 ± 2043.6	1956.6 ± 1157.9	1938.8 ± 174.0	2091.3 ± 1095.6	3292.3 ± 42.5	3052.2 ± 2.9
Pentacosane (C25)	2316.1 ± 670.3	1727.3 ± 46.6	1110.2 ± 39.1	1380.1 ± 208.2	1618.0 ± 58.2	1459.4 ± 41.7
Heptacosane (C27)	9922.9 ± 2033.6	7568.1 ± 1210.8	5648.9 ± 286.4	6599.2 ± 217.7	3657.8 ± 3375.8	2893.2 ± 2622.9
Pentadecane, 8-hexyl	656.2 ± 186.1	437.9 ± 68.9	400.9 ± 60.0	394.7 ± 118.2	3729.7 ± 3157.1	3627.8 ± 3120.5
Hexacosane, 9-octyl	784.3 ± 222.3	528.6 ± 45.9	407.1 ± 14.7	427.4 ± 37.1	542.0 ± 1.8	448.7 ± 0.3
Octacosane (C28)	1383.1 ± 312.5	1124.8 ± 101.5	856.5 ± 0.9	1006.3 ± 5.2	790.1 ± 252.4	684.5 ± 165.4
Nonacosanol	1185.3 ± 566.0	864.3 ± 133.4	392.4 ± 168.9	312.8 ± 26.5	654.5 ± 44.2	679.9 ± 47.2
Nonacosane (C29)	16,101.1 ± 3245.7	13,055.1 ± 1876.3	11,667.5 ± 345.1	13,580.3 ± 605.4	14,648.6 ± 202.6	12,125.0 ± 1379.7
Methyl, nonacosane	3628.8 ± 792.4	2914.8 ± 242.3	1792.9 ± 66.6	1953.8 ± 621.0	2784.3 ± 7.5	2074.7 ± 137.8
Hentriacontane (C31)	3426.1 ± 804.4	2851.5 ± 290.4	2501.8 ± 61.7	2902.7 ± 223.3	3262.4 ± 58.4	2713.3 ± 254.2
Methyl-hentriacontane	8454.6 ± 1864.5	6941.6 ± 763.2	4528.1 ± 260.2	4877.7 ± 1531.3	6810.8 ± 74.4	5075.7 ± 230.2
Tritriacontane (C33)	465.4 ± 167.6	496.3 ± 122.4	294.2 ± 16.0	357.0 ± 25.6	395.5 ± 14.9	335.4 ± 13.5
Tetratriacontane (C34)	246.5 ± 22.5	**114.5** ± **114.5**	ND ± ND	ND ± ND	ND ± ND	ND ± ND
Pentatriacontane (C35)	443.0 ± 171.7	**335.8** ± **335.8**	ND ± ND	ND ± ND	ND ± ND	ND ± ND
Heptatriacontane (C37)	ND ± ND	**175.7** ± **175.7**	ND ± ND	ND ± ND	ND ± ND	ND ± ND
Total	56,898.5 ± 13,833.9	45,574.1 ± 62.8	33,610.7 ± 56.0	38,583.4 ± 6193.3	46,417.4 ± 522.3	37,599.0 ± 2046.0

Mean values of two experiments performed in triplicates.

*SE* standard error, *ND* not detected.

Bold—CHC detected only in one experiment.

Although the WS mosquitoes presented 18 out of 19 CHCs, they had 1.25-fold more content than BS mosquitoes. This also happened with the WPb mosquitoes, with 1.23-fold more quantity than the BPb mosquitoes. In contrast, BB mosquitoes had 1.15-fold more than the WB mosquitoes (Table 1, Fig. 1B). Despite these inter phenotype differences, the CHCs content of both White and Brown mosquitoes behaved almost similarly in response to the feeding/challenged status in terms of the quantity of each CHC. In general, compared to the sugar diet, the total CHCs content decreased after the blood-feeding and parasite challenge. However, if the blood contained *Plasmodium* ookinetes, the total content of CHCs increased in the White phenotype while remained the same in the Brown phenotype when compared to their respective blood-fed groups (Table 1, Fig. 1B). This increment in CHC content is especially the case for pentadecane 8-hexyl, which increased almost tenfold after the *Plasmodium* challenge (Fig. 1B). An exception of this general trend was the heptacosane (C27), which showed a twofold reduction after the *Plasmodium* challenge.

A principal component analysis (PCA) was then carried out to determine the influence of the individual CHCs on the overall differences observed between the phenotypes and feeding treatments. It was found that nonacosane (C29), methyl-hentraicontane, heptacosane (C27), and to a lesser extent pentadecane 8-hexyl, Z-12 penstacosene, and hentriacontane (C31), were responsible for explaining the greatest amount of the variation (Fig. 1D). That is, these CHCs account for the differences found between the phenotypes and feeding treatments. To further dissect these differences and establish what kind of variation they explain, a hierarchical cluster analysis was performed on the PCA factor scores to find the dissimilarities between the phenotypes and feeding treatments. The resulting dendrogram clustered the groups according to their feeding or challenged status (Fig. 1E), meaning that both phenotypes responded more similar to the diet than the differences they have between each other. Thus, the susceptibility/resistance to *Plasmodium* can only be determined if the feeding or challenged status is known beforehand.

### The CHCs are modified differentially between the phenotypes after a *Plasmodium* challenge

**Figure 2:**
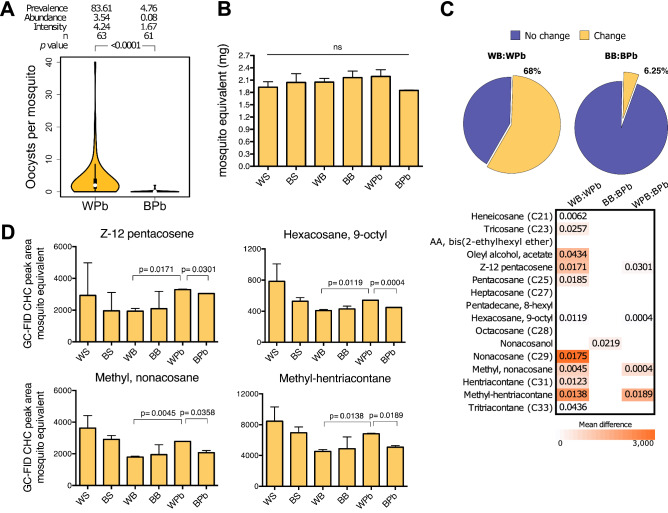
*P. berghei* infection alters the CHC composition. (**A**) Infection parameters of the White-susceptible and Brown-resistant phenotypes fed with infected blood (WPb; BPb, respectively). White and Brown females 5 (dpe) were challenged with 900 ookinetes per microliter of a *P. berghei* strain that constitutively expresses the GFP protein. Three days post-challenge, the midguts were dissected, and the abundance, intensity and prevalence of the infection were determined by fluorescence microscopy. Results of two independent experiments with approximately n = 30 mosquitoes for each group. Data was analyzed with the Mann–Whitney U test. (**B**) Mosquito-equivalent mass (mass/50 mosquitoes) of the White and Brown phenotypes with different diets based on 8% sucrose solution (WS; BS), blood (WB; BB), and infected blood (WPb; BPb). Three days post-feeding, the mass of 50 mosquitoes per group was measured to extract the cuticular hydrocarbons afterwards. Representation of the mean ± standard error of the mean (SEM) of three independent experiments. (**C**) Percentage of CHCs that changed significantly between the blood-fed and infected blood-fed mosquitoes (pie charts of White blood vs. White Pb-challenged, left; and of Brown blood vs Brown Pb-challenged, right). Heat map showing which CHCs changed significantly between the blood-fed and Pb-challenged mosquitoes per phenotype and between phenotypes for the challenged condition. The colour gradient represents the mean difference of the CHC GC-FID area of mosquito extracts between the different conditions (WPb:WB, BPb:BB, and WPb:BPb). Only compounds with significant differences were represented with colour, white boxes represent no significant change. The numbers in the boxes are the *p*-values calculated by a two-tailed t test of two independent experiments performed in triplicates. (**D**) CHCs with significant changes between the susceptible and resistant phenotypes fed with different diets and *Plasmodium*-challenged. GC-FID lipid area of mosquito extracts of the two phenotypes with diets based on 8% sucrose (WS; BS), blood (WB; BB), and infected blood (WPb; BPb). Mean ± SEM of two independent experiments performed in triplicates each with 50 mosquitoes per group. *p*-values of pairwise two-tailed t tests.

Although the main differences in the CHCs were found between the feeding treatments, the White and Brown challenged mosquitoes also had differences that are worth exploring further. Figure 2A depicts the infection parameters of both phenotypes, showing that the Brown mosquitoes eliminated the parasites almost totally. On the contrary, 84% of the White mosquitoes could not resolve the infection, having on average four oocysts per mosquito. The Brown challenged mosquitoes weighted 0.31 ± 0.16 mg less than the Brown blood-fed mosquitoes and 0.34 ± 0.17 mg less than the White challenged mosquitoes (Fig. 2B). Albeit non-significant, this is a reduction of 16% in the bodyweight, suggesting a diversion of nutrients to contend against the parasite. We have shown previously that the Brown mosquitoes present an intense anti-*Plasmodium* response during the ookinete invasion that is consistent with the low infection prevalence and intensity^38^. It seems that this response is costly and might require different nutrient management to sustain the over expression of immune factors.

The fact that 95% of the challenged Brown mosquitoes did not become infected can also explain why only 6.25% of the CHCs showed quantitative differences relative to the blood-fed mosquitoes (Fig. 2C). In contrast, the White phenotype, with a high prevalence of infection, showed quantitative differences in 68% of the CHCs after the *Plasmodium* challenge with respect to the blood-fed mosquitoes (Fig. 2C). Here, the presence of parasites has to be considered, since it has been shown that infected mosquitoes up-regulate the lipophorins^40^, indicating that the parasite is causing the differential changes observed between the phenotypes to some extent. This result further explains why the total CHC content increases after an infected blood meal in the White mosquitoes compared to the uninfected blood meal group, whereas in the Brown mosquitoes, which did not become infected, this increase in the total CHC content does not take place (Fig. 1B). On the same note, the CHC changes observed in BPb compared to BB may be solely due to physiological changes associated with the activation of an effective immune response against the parasite.

A comparison between White and Brown mosquitoes after the *Plasmodium* challenge showed changes only in four CHCs (Fig. 2C); namely, Z-12 pentacosene, hexacosane 9-octyl, methyl-nonacosane, and methyl-hentriacontane, which are also included among the CHCs that changed significantly between the blood-fed and *Plasmodium*-challenged White mosquitoes (Fig. 2D). On the other hand, nonacosanol was the only CHC that changed in the Brown phenotype upon the parasite challenge, a change that is not shared with the White phenotype.

## Discussion

Many physiological aspects that impact on lifespan, general health, and reproductive potential are deeply affected by the dietary composition^1,9,41^. This especially holds true for mosquitoes, which need a copious protein-rich blood meal to reproduce. Notwithstanding, this is also a source of infections for mosquitoes and for the humans they feed on. Therefore, understanding the mosquito physiology concerning their feeding requirements and the eventual interaction with parasites, may give clues on how to block the transmission of vector-borne diseases. In this work, we showed that the dietary composition affects the CHC profile in *An. albimanus.* This makes it possible to detect the mosquitoes’ dietary status and, most importantly, to detect mosquitoes that have fed on a *Plasmodium-*infected blood meal by the determination the CHC profile. It is noteworthy that Brown mosquitoes were classified together with the White mosquitoes (Fig. 1E) even though only 5% contained parasites, meaning that it is possible to distinguish not only infected mosquitoes but those who have resolved the infection also. The determination of the CHC profile by GC brings several advantages in comparison to other techniques, like midgut or salivary gland dissection, or genomic analysis. The most significant advantage is that the mosquitoes do not need preservation or a cold chain to be analyzed, as the mosquitoes can be damaged, dried, or even old (pinned museum specimens, for example)^17^. Additionally, the CHC extraction with hexane is non-destructive, so mosquitoes can still be used afterwards.

The determination of the causes or effects of the observed changes in the CHC composition is a more challenging task and requires further experimentation. However, the results presented herein provide the basis for establishing new hypothesis that will deepen our understanding of the general mosquito metabolism after a blood-feeding and parasite challenge. The sugar diet represents an abundant source of carbons that is reflected in the higher production of CHCs, and the presence of longer CHCs. Whether infected or not, the blood caused the total amount and composition of the CHCs to decrease, especially for the very long-chain CHCs, which may indicate a shortage of carbons and an inhibition/limitation of the fatty acid synthases or elongases activity^2^. Previous studies in *Drosophila melanogaster* reported similar results, where a diet based on yeast or sugar caused the CHC composition to change in opposite directions^41^. Fedina et al.^41^ reported that a yeast-based diet reduced the relative abundance of the CHCs, while a sugar-based diet increased the relative abundance. In this regard, one of the mechanisms by which mosquitoes acquire resistance to insecticides is by limiting the penetration of chemicals through the thickening and alteration of the cuticle composition^4,24,28^. In the case of *An. albimanus* this is interesting in two ways: first, the White mosquitoes have been shown to be resistant against insecticides, and although it is not known why, it is possible that the higher amount of CHCs in comparison to Brown mosquitoes may play a role in such phenomenon. And second, following the same argument, a blood meal may cause the mosquitoes to become more susceptible to insecticides due to the reduction in the CHC content and composition.

In anopheline mosquitoes, the CHC profile has been shown to change progressively on a daily basis, allowing age-grading mosquitoes with a 1-day-old difference^14^. This aspect has been used to determine the probability for a female to be old enough to transmit the *Plasmodium* parasite^23^. The changes in the CHC profiles we observed after three days of the different diet feedings have to be considered if age-grading is meant to be used with field mosquitoes. If we consider that the CHC profile of the sugar-fed mosquitoes portrays a basal state, the 30% reduction in the CHC content in the White blood-fed mosquitoes and the 16% reduction in the Brown blood-fed mosquitoes, represent a drastic change in the CHC content in a short period of time that has to be accounted for in this kind of studies.

An interesting observation was the sizeable weight loss in the Brown-resistant phenotype when exposed to *P. berghei*. The strong transcriptional response of immune markers of the Brown phenotype during *P. berghei* invasion^38^, could imply energetically costly metabolic shifts that may be responsible for the observed 16% weight loss in this phenotype^42,43^. In *D. melanogaster,* it has long been recognized that the activation of immune cells leads to a metabolic switch associated with increased energy consumption^44^. The contrasting effects of an infected blood meal on the CHC composition between the White and Brown phenotypes, taking as reference their respective uninfected blood meal groups, emerge due to the interaction between the vector and the parasite but as a result of different scenarios. In the Brown mosquitoes we have a scenario where there are no parasites and a strong immune response, and in the White mosquitoes a scenario where there are parasites and a lack of an effective immune response. The overall balance is that Brown mosquitoes spend more energy while trying to maintain homeostasis at the expense of losing weight, while the white mosquitoes actually tend to gain a few micrograms of weight and CHCs.

Besides its importance in the adaptive responses to the environment, the CHCs also function as attractiveness signals for mating purposes. It has been hypothesized that the ~ 40% reduction in heneicosane (C21) and tricosane (C23) in *An. gambiae* females after mating reduce their attractiveness to courting males, reducing mating in already inseminated females^22^. In our experiments, females at 5 dpe are already inseminated, so that the heneicosane content may already be reduced compared to unmated females. However, when blood-fed, the abundance of heneicosane was reduced to undetectable levels, which may indicate the males that these females are even less prone to mating. Remarkably, in the *Plasmodium*-challenged mosquitoes, heneicosane (C21) is present at almost the same abundance as the sugar-fed females. This likely causes the *Plasmodium*-challenged females to still respond to males as if they had not been blood fed. Albeit a trade-off between reproduction and immunity has not been determined in *An. albimanus*, as has been shown for *An. stephensi*^45^, the presence of heneicosane in the challenged mosquitoes might indicate that such tradeoffs also occur in *An. albimanus*.

We identified four cuticular components with significant differences between the susceptible and resistant phenotypes to *P. berghei* infection (Fig. 2D): Z-12 pentacosane, hexacosane 9-octyl, methyl-nonacosane, and methyl-hentriacontane. Comparative analyzes in multiple species of insects have revealed that these CHCs have evolved to perform a number of functions in chemical communication, with special emphasis on their reproductive biology^3,10^. Studies on *Z*-12 pentacosane reveal that it is a potent oviposition pheromone^46^. Similarly, hexacosane, 9-octyl functions as a sex attractant pheromone^47^, and nestmate recognition^48^. Meanwhile, methyl-nonacosane has been identified to have a role in fertility signalling^49^ and behavior^50^. Several studies exist for the role of methyl-hentriacontane, and has been proposed to be part of the phenotypic variability^51^ and behavior^50^. However, the specific contribution of these CHCs in mosquitoes is not clear, so these molecules should be analyzed to investigate their potential role in the behaviour of infected mosquitoes.

Interestingly, pentadecane, 8-hexyl tends to increase in both strains only when an infection is involved (Fig. 1B, Table 1). This compound (along with octacosane and triacontane, also detected in *An. albimanus*’ cuticle) is present in extracts from green seaweed, and has been implicated in antibacterial and fish pathogen functions^52^. It will be interesting to examine whether this molecule exerts an anti-*Plasmodium* activity.

The blood meal represents the principal source of proteins and lipids for the mosquito, which are firstly metabolized in the midgut. Here, cholesterol, phosphatidyl choline, phosphatidyl ethanolamine, cholesteryl ester, and diacylglyceride are the major lipoprotein-associated lipids^53^. The mosquito cell's common lipid uptake pathway relies on the lipophorin complex of proteins^53^. These proteins are present in the hemolymph and bind to lipids, carrying them as a reusable shuttle to or from the fat body and other organs for metabolism and storage. Lipophorins have been shown to be upregulated after a *Plasmodium* infection^40^, where the knockdown of lipophorins led to a strong restriction in oocysts numbers by 90% in *Ae. aegypti*^40^ and of fourfold in *An. gambiae*^54^. Since *Plasmodium* partially lacks the genes required for lipid synthesis, the parasite hijacks these lipids from the lipophorins for its growth, increasing its infectivity, virulence, and quantity of sporozoites^55^. Thus, the mosquito lipid metabolism is crucial for *Plasmodium* development, especially during sporogony, where the oocysts increase in size about tenfold producing thousands of sporozoites, each with its own lipid membrane^30^.

Free aminoacids obtained through diet such as valine, isoleucine and methionine are substrate for the synthesis of propionyl-CoA by the mosquito, which in conjunction with malonyl-CoA, are incorporated to hydrocarbons to form methyl-branched hydrocarbons^10^. The CHCs are synthesized from acetyl-CoA in an elongation reaction to form a long-chained fatty acyl-CoA in the oenocytes^2^. Then P450 decarbonylase converts them to long-chained hydrocarbons that are transported to the cuticle. The first studies that addressed the effects of *Plasmodium* infection on the physiology of mosquitoes reported that the contents of valine, isoleucine, methionine, histidine and lysine in the hemolymph of infected mosquitoes markedly decreases^56,57^, while their midguts use eight times as much glucose^58^. Other effects of *Plasmodium* infection on mosquitoes are changes in their behaviour. The infected mosquitoes have increased probing time, more persistent in biting, increased frequency of multiple feedings, and greater feeding-associated mortality^43^. In *An. albimanus*, the infection with *P. berghei* lead to a differential expression of proteins in the infected midgut^59^ and an increase in the concentration of brain proteins involved in the cellular metabolic pathway and neural function^60^.

Here, we address whether the CHC composition of the malaria vector *An. albimanus* is altered after blood-feeding and parasitic infection, finding that indeed the CHC composition changes dependently on these factors. Therefore, the CHCs bear close relationship with the mosquito physiology and in particular with important aspects of the adaptive responses against the environment. It is interesting that multiple CHCs that changed upon infection are associated with the reproductive biology of insects, indicating that a *Plasmodium* infection may be associated with behavioural changes or the physiology of the gonotrophic cycle in *An. albimanus*. Malaria is the most important vector-borne disease in terms of morbidity and mortality worldwide, this work provides new information to further investigate the causes and implications of the susceptible/resistance condition against insecticides and parasites. The susceptibility/resistance is multi-factorial phenomenon that impacts on all aspects of an organism, and if a certain aspect is not affected, usually there are compensations with other physiological systems. We are still lacking a view of the whole picture for this phenomenon, but studies like the one we are presenting here are letting us sharpen the picture pixel by pixel. Perhaps more interesting is the potential for developing technologies to control the mosquito population, or prevent disease transmission by understanding how the mosquito changes as a whole after a blood-meal, especially if the blood is infected with parasites.

## Methods

### Mosquito handling

*An. albimanus* White and Brown phenotypes were derived from the parental Tapachula strain and were reared at 28 °C under 70–80% humidity and a 12/12 h day/night cycle in the insectary of the INSP, Cuernavaca Morelos. *An. albimanus* phenotype selection was carried out in the pupal stage, discriminating against the presence of a white stripe in the dorsal side surrounded by the brownish to the grey coloration of the rest of the body (White) or by the absence of this stripe (Brown).

Groups of 150 female mosquitoes were separated to form the following experimental treatment groups with 50 females per group: White + sugar (WS), Brown + sugar (BS), White + blood (WB), Brow + blood (BB), White + *P. berghei* (WPb) and Brown + *P. berghei* (BPb). Female mosquitoes were fed *ad-libitum* with sterile cotton balls dampened with maintenance solution (8% sucrose (Molecular Probes), 0.05% para-aminobenzoic acid (Sigma) and penicillin, streptomycin and neomicin (PSN, Sigma). The dampened cotton balls were replaced every 24 h. All methods were carried out in accordance with relevant guidelines and regulations. The experiments were approved by the Biosafety and Ethics Committees of the Instituto Nacional de Salud Pública (INSP, Mexico) and we performed following the ARRIVE guidelines.

### *Plasmodium berghei* ookinete culture and mosquito feedings

Ookinete culture was carried out as described in Recio-Tótoro et al.^61^. Briefly, 6–8 weeks old male BALB/c mice were treated with 200 µl of phenylhydrazine (6 mg/ml in saline) intraperitoneally (IP) two days before the IP inoculation of 2–4 × 10^8^ GFP-expressing parasites. When the parasitemia reached 15–25%, and after gametocyte viability verification, the infected blood was extracted by cardiac puncture with a heparinized syringe from the CO_2_ euthanized mice and incubated at 19–20 °C for 18–20 h in RPMI-Ook medium (1:4, blood medium) to allow ookinete formation. The ookinetes were counted using a Neubauer chamber, and the culture was centrifuged at 2000 rpm for 5 min to resuspend the pellet in foetal calf serum (FCB, Hyclone) at a density of 900 ookinetes per microlitre.

Mosquito feedings were carried out by the Standard Membrane Feeding Assay, at 5 dpe, using GFP-expressing ookinete cultures or uninfected-blood treated as for the ookinete cultures. Sugar cotton pads were removed 4 h prior blood feeding to ensure engorgement. Feeders were left in contact with the mosquitoes for 30–60 min at 37 °C using a water re-circulator. After feeding, mosquitoes were left without cotton pads overnight to eliminate partially or non-engorged females by dehydration. Afterwards mosquitoes were kept at 19–20 °C and fed on sterile cotton pads dampened with maintenance sugar solution *ad-libitum* until CHC extraction and infection quantification.

### Prevalence, abundance, and intensity of infections

To assess the parasite infections, the midguts of 30 females were dissected in PBS, mounted on microscopy slides, and observed under an epifluorescence microscope (Leica DM1000 microscope with a mercury lamp and the Leica H3 filter cube). *P. berghei*-GFP oocysts were counted, and the prevalence, abundance, and intensity of infections were determined in two independent experiments. The infection parameters were calculated as follows: prevalence = percentage of infected mosquitoes; intensity = average number of oocysts per infected mosquito; abundance = average number of oocysts per total mosquitoes.

### Lipid extraction and gas chromatography-mass spectrometry/-flame ionization analysis

Two independent experiments were performed with three replicates each. Groups of 50 females of 8 dpe of each treatment (sucrose, blood and *P. berghei*) were anesthetized at 4 °C for 15 min. Mosquitoes’ weight was measured using an analytical scale (Gibco). For each experiment, CHCs were extracted by hexane immersion; 50 females were placed in 1.5 ml amber-crystal vials and incubated with 1 ml of hexane (Merck; HPLC grade) for 15 min at room temperature. The resulting liquid was transferred to a new vial and gently evaporated under an N_2_ stream. The samples were then analyzed by gas chromatography coupled to either mass spectrometry or flame ionization detectors.

For the qualitative identification of hydrocarbons, the samples were resuspended in 10 µl of hexane, and aliquots of 1 μl of extract samples were analysed using a 6890 N gas chromatograph (GC, Agilent Technologies, Inc., Santa Clara, CA, USA), coupled with an EI-quadrupole 5973 mass spectrometer (MS, Agilent Technologies). The GC was equipped with a ZB-5MS capillary column (Zebron Phenomenex, Inc., Torrance, CA, USA; 30 m × 0.25 mm, with a 0.25 µm phase coating thickness) and the carrier gas was ultra-high purity helium with a column head pressure of 10 psi. Oven was set at 60 °C for 3 min, then the temperature was increased from 60 to 110 °C at 25 °C/min rate and from 110 to 310 °C at 5 °C/min rate. The final temperature was maintained for 20 min. The transfer line was at 290 °C. Splitless injection (3 min hold) was performed using an automated sampler with an injector temperature of 280 °C. Mass spectral data were acquired in full scan mode over a range of 40–600 m/z. Integration of chromatograms and the analysis of hydrocarbon was made following the procedures described by Witek et al.^62^. The quantitative analysis of epicuticular hydrocarbons was performed by GC coupled to a Flame Ionization Detector (FID) by using a Shimadzu gas chromatograph (GC-2010 Plus) at the same conditions and using the same column as described above. Quantification was performed in three technical replicates based on the use of both heptadecane as internal standard and by external standard curves obtained with pure alkane standards (Sigma, Milan, Italy).

### Statistical analysis

PCA and dendrogram analysis, as well as the evaluation of the mosquito infections, were carried out in R version 4.0.2. The *prcomp* function was used for the PCA and plotted using the factoextra package. The cluster dendrogram was obtained from the PCA matrix using the mosquito feeding treatments as groups, obtaining the distance matrix with the *dist* function, and the dissimilarity structure with the *hclust* function using the complete linkage method. The number of oocysts per mosquito was plotted using the vioplot package and analyzed with a Mann–Whitney U test. Student’s t-test comparisons were carried out in Prism 6. Pie charts were plotted in Gnumeric.

### Ethical review

Along with ARRIVE guidelines, all the methods were carried out in accordance with relevant guidelines and regulations. This study was approved by the Biosafety and Ethics Committees of the Instituto Nacional de Salud Pública (INSP, Mexico).
